# Sodium-Glucose Cotransporter 2 (SGLT2) Inhibitors in Non-diabetic Heart Failure: A Systematic Review of Randomized Controlled Trials and Real-World Evidence

**DOI:** 10.7759/cureus.90684

**Published:** 2025-08-21

**Authors:** Mohammed O Ibrahim, Osama M Mohamed, Nagwa M Ali

**Affiliations:** 1 College of Medicine, Mohammed Bin Rashid University of Medicine and Health Sciences, Dubai, ARE; 2 Family Medicine, SEHA Clinics, Abu Dhabi Health Services Company (SEHA), Abu Dhabi, ARE; 3 Rheumatology, Mediclinic Airport Road Hospital, Abu Dhabi, ARE

**Keywords:** dapagliflozin, empagliflozin, heart failure, heart failure with preserved ejection fraction, heart failure with reduced ejection fraction, non-diabetic patients, sglt2 inhibitors, sodium-glucose cotransporter-2 (sglt2) inhibitors, systematic review

## Abstract

Sodium-glucose cotransporter 2 inhibitors (SGLT2i) were initially developed to improve glycemic control in patients with type 2 diabetes. More recently, growing evidence has highlighted their cardiovascular benefits, particularly in patients with heart failure (HF), irrespective of diabetic status. This systematic review evaluates the efficacy and safety of SGLT2i, including dapagliflozin and empagliflozin, in non-diabetic patients with HF across different phenotypes, namely heart failure with reduced ejection fraction (HFrEF) and preserved ejection fraction (HFpEF).

A comprehensive literature search was conducted in PubMed, Scopus, and Embase for studies published between January 1, 2015, and January 15, 2024, in accordance with Preferred Reporting Items for Systematic reviews and Meta-Analyses (PRISMA) guidelines. Eligible studies included adult patients with HF, reported outcomes for non-diabetic subgroups, and assessed treatment with SGLT2i. Six studies met the inclusion criteria: four randomized controlled trials (RCTs), one pooled patient-level analysis, and one observational cohort study involving 16,264 non-diabetic patients. A pooled patient-level analysis incorporated data from DAPA-HF and DELIVER; to avoid duplication, only DAPA-HF was analyzed individually, while DELIVER was included solely within the pooled dataset.

SGLT2i use was associated with lower cardiovascular mortality (hazard ratio (HR): 0.73-0.86) and reduced HF hospitalization, with consistent benefits in both HFrEF and HFpEF. Adverse events such as hypoglycemia, volume depletion, and infections were uncommon and typically mild, although genital infections were more frequent than placebo, and severe adverse events were not increased. Due to heterogeneity in study designs, outcome definitions, and HF phenotypes, quantitative meta-analysis was not feasible. A structured qualitative synthesis was performed.

To our knowledge, this is one of the first systematic reviews to comprehensively evaluate the use of SGLT2i in non-diabetic patients with HF across phenotypes. The findings reinforce the role of SGLT2i as a foundational therapy in non-diabetic HF and highlight the need for broader implementation in practice.

## Introduction and background

Heart failure (HF) affects over 64 million people globally and remains a leading cause of hospitalization, disability, and mortality, particularly among older adults [1]. HF is commonly classified as heart failure with reduced ejection fraction (HFrEF) or preserved ejection fraction (HFpEF), with both subtypes contributing substantially to global disease burden [1]. While current pharmacologic therapies, including beta-blockers, angiotensin-converting enzyme (ACE) inhibitors, angiotensin receptor-neprilysin inhibitors (ARNIs), and mineralocorticoid receptor antagonists (MRAs), have led to measurable improvements in outcomes, many patients still experience recurrent hospitalizations, persistent symptoms, and reduced quality of life [2-4].

Sodium-glucose cotransporter 2 inhibitors (SGLT2i) were originally developed to lower blood glucose by promoting urinary glucose excretion in patients with type 2 diabetes mellitus [5]. However, landmark cardiovascular (CV) outcome trials, such as EMPA-REG OUTCOME, CANVAS, and DECLARE-TIMI 58, unexpectedly revealed significant reductions in HF hospitalizations and CV mortality, benefits that appeared to be independent of glycemic control [6-8]. These findings sparked interest in evaluating the effects of SGLT2i in patients with HF regardless of diabetes status, particularly as real-world evidence and subgroup analyses began to highlight consistent outcomes among non-diabetic individuals.

Two major randomized controlled trials (RCTs), DAPA-HF and EMPEROR-Reduced, investigated the use of dapagliflozin and empagliflozin in patients with HFrEF. Notably, more than half of the participants in both trials were non-diabetic. DAPA-HF demonstrated a 26% relative risk reduction in the composite outcome of worsening HF or CV death, while EMPEROR-Reduced reported a hazard ratio (HR) of 0.75 for CV death or HF hospitalization, with consistent benefits observed among non-diabetic participants [9,10]. These subgroup analyses were pre-specified, thereby strengthening the reliability of the findings for this population [9,10].

Building on this evidence, the EMPEROR-Preserved trial became the first randomized trial to demonstrate the benefit of SGLT2i in patients with HFpEF, a population that had historically lacked effective therapies [11]. Once again, the observed advantages were observed in patients without diabetes, supporting the idea that the cardioprotective properties of SGLT2i are essentially independent of their action in lowering glucose levels.

SGLT2i confer CV benefit through multiple interrelated physiological pathways. Among these, their capacity to induce natriuresis and osmotic diuresis leads to effective plasma volume modulation, which diminishes both cardiac preload and afterload - mechanisms fundamental to the management of HF [12,13]. Additionally, they improve myocardial energetics through enhanced ketone body metabolism, reduce myocardial fibrosis and inflammation, protect renal function, and potentially modulate neurohormonal activity, all of which are relevant to HF pathophysiology regardless of glycemic status [14,15]. This range of mechanisms can be broadly grouped into hemodynamic, metabolic, renal, and neurohormonal domains [12-15]. Although rare in trials, emerging safety concerns such as euglycemic ketoacidosis have prompted interest in better understanding adverse event profiles in non-diabetic populations [14,15].

Observational evidence supports these findings. Large-scale observational studies, including the CVD-REAL Nordic registry and EMPRISE, have shown decreased hospitalizations and improved renal outcomes in HF patients receiving SGLT2i. However, these studies did not report outcomes stratified by diabetes status and were therefore not eligible for inclusion in our analysis [16,17].

The clinical adoption of SGLT2i in non-diabetic HF patients remains suboptimal, despite the increasing evidence supporting their use. Numerous clinicians primarily link this drug class to glucose regulation, which results in its underprescription among eligible non-diabetic patients. Additional barriers may include concerns about potential adverse effects, limited provider familiarity, and uncertainties regarding cost-effectiveness and insurance coverage [18]. Although precise estimates vary, real-world data suggest that SGLT2i uptake among eligible non-diabetic HF patients may remain below 30% [18]. However, updated guidelines from the American Heart Association (AHA), American College of Cardiology (ACC), and European Society of Cardiology (ESC) now recommend the use of SGLT2i in patients with HFrEF regardless of diabetic status [4,19].

Still, important knowledge gaps remain. Most large trials were not specifically powered for non-diabetic subgroups, and although subgroup analyses suggest comparable benefits in diabetic and non-diabetic patients, definitive conclusions are limited by statistical power. Moreover, data on non-diabetic patients with HFpEF and advanced chronic kidney disease (CKD) are less robust, and ongoing trials are expected to address these underrepresented populations. Additionally, long-term safety data and cost-effectiveness studies specifically targeting non-diabetic HF populations remain limited.

To our knowledge, this is one of the first systematic reviews to systematically synthesize evidence from both RCTs and real-world studies specifically evaluating the efficacy, safety, and clinical applicability of SGLT2 inhibitors in non-diabetic patients with HF across different phenotypes. Previous reviews have primarily focused on diabetic populations or mixed cohorts, without a dedicated analysis of non-diabetic subgroups.

In light of these considerations, we conducted a systematic review to synthesize the current clinical data on the use of SGLT2i in adult non-diabetic HF patients. The objective is to describe and clarify their role across HF subtypes, assess safety outcomes, and inform broader implementation in this undertreated and underrecognized population.

## Review

Methods

Protocol and Reporting Guidelines

This systematic review was conducted in accordance with the Preferred Reporting Items for Systematic Reviews and Meta-Analyses (PRISMA) 2020 guidelines [20,21]. The review protocol was not prospectively registered due to time constraints during project initiation. While this limits protocol transparency and may introduce risk of selective reporting, all decisions regarding eligibility, synthesis, and bias assessment were predefined and reported in adherence with PRISMA. The methodology was designed to follow established best practices for qualitative evidence synthesis, including structured study selection, independent screening, and transparent data extraction procedures.

Objectives

This systematic review aimed to evaluate the effects of SGLT2i on CV outcomes and safety in non-diabetic adults with HF. This study aimed to assess the impact on CV mortality, all-cause mortality, HF hospitalization, and adverse events in populations with HFrEF and HFpEF.

Eligibility Criteria

Studies were eligible for inclusion if they met the following criteria: they enrolled both diabetic and non-diabetic patients and reported outcomes specifically for the non-diabetic subgroup; intervention involving any SGLT2i (including dapagliflozin, empagliflozin, canagliflozin, or sotagliflozin); the comparator was placebo, usual care, or other standard therapy; and reported outcomes including CV mortality, all-cause mortality, or hospitalization for HF. Eligible study designs include RCTs, post hoc subgroup analyses, and observational cohort studies published in English. Studies published in languages other than English were excluded due to a lack of translation resources, which may introduce language bias. Studies were excluded if they lacked data specific to non-diabetic subgroups, focused solely on type 1 diabetes, or were reviews, editorials, or conference abstracts without original data. Gray literature and preprints were not included in this review, which may limit the retrieval of unpublished findings; however, formal registries such as ClinicalTrials.gov were searched to identify ongoing studies. Ongoing or unpublished trials identified in registries were recorded but excluded from synthesis if no results were available.

Data Sources and Search Strategy

A comprehensive literature search was conducted using PubMed, Embase, Cochrane Central Register of Controlled Trials (CENTRAL), and ClinicalTrials.gov to identify relevant studies published between January 1, 2015, and January 15, 2024. The search period was chosen to encompass the timeframe during which SGLT2i were first investigated for CV and HF outcomes beyond glycemic control. Search terms included combinations of keywords and Medical Subject Headings (MeSH) such as: "SGLT2 inhibitors," "dapagliflozin," "empagliflozin," "heart failure," "HFrEF," "HFpEF," "non-diabetic," "mortality," and "hospitalization." The full electronic search strategy for PubMed has been provided in Appendix A. In addition, reference lists of all included studies and relevant reviews were manually screened to identify any relevant articles that may have been missed during the electronic database search.

Study Selection and Data Extraction

Two independent reviewers screened titles and abstracts, followed by a full-text review of potentially eligible studies. Duplicate records were removed using reference management software before screening began. Relevant data were extracted independently, including study design, sample size (overall and non-diabetic subgroup where applicable), intervention, comparator, follow-up duration, and reported outcomes. Discrepancies during study selection or data extraction were resolved by consensus; if disagreement persisted, a third reviewer arbitrated. The same two reviewers conducted bias assessment and extraction without blinding to the study authors.

Risk of Bias Assessment

Risk of bias was assessed independently for each included study. RCTs were evaluated using the Cochrane Risk of Bias 2.0 (RoB 2.0) tool (The Cochrane Collaboration, Oxford, UK), which considers domains such as the randomization process, deviations from intended interventions, missing outcome data, measurement of the outcome, and selection of the reported result [22]. The only included observational study was assessed using the Newcastle-Ottawa Scale (NOS), a validated tool for evaluating non-randomized studies in meta-analyses, focusing on selection, comparability, and outcome domains [23]. The NOS assigns a maximum score of 9 points, with higher scores indicating better methodological quality: 7-9 points reflect low risk of bias, 4-6 points indicate moderate risk, and 0-3 points suggest high risk of bias. The risk-of-bias assessments for all included studies are summarized in Tables 1, 2. Although the Grading of Recommendations, Assessment, Development, and Evaluation (GRADE) framework was not formally applied, the quality of evidence and study limitations were considered narratively. Risk-of-bias results did not formally alter study weighting but informed interpretation of the findings.

**Table 1:** Cochrane RoB 2.0 Domain-Level Risk of Bias Assessment for Included RCTs RCT: randomized controlled trial; RoB: risk of bias

Study	Randomization Process	Deviations From Intended Interventions	Missing Outcome Data	Outcome Measurement	Selection of the Reported Result	Overall RoB
DAPA-HF [9]	Low	Low	Low	Low	Low	Low
EMPEROR-Reduced [10]	Low	Low	Low	Low	Low	Low
EMPEROR-Preserved [11]	Low	Low	Low	Low	Low	Low
Santos-Gallego et al. [24]	Low	Some concerns	Low	Low	Some concerns	Moderate
Jhund et al. [25]	Low	Low	Low	Low	Low	Low

**Table 2:** Newcastle-Ottawa Scale (NOS) Risk of Bias Assessment for Svanström et al. [26] HF: heart failure; each asterisk (*) indicates that the criterion for that domain was fulfilled.

Domain	Criteria	Score (*)
Selection (max. 4*)		
Representativeness	Truly representative of the average HF patient in the community	*
Selection of the cohort	Drawn from a secure national registry	*
Ascertainment of exposure	Prescription records in the national database	*
Demonstration of outcome absence at start	No HF hospitalization at cohort entry	*
Comparability (max. 2*)		
Control for confounders	Adjusted for age, sex, comorbidities, and medications	**
Outcome (max. 3*)		
Outcome assessment	Hospital records and the national death registry	*
Follow-up duration	≥12 months	*
Adequacy of follow-up	Minimal loss to follow-up due to national data linkage	*
Total Score		9/9

Data Synthesis

Due to clinical and methodological heterogeneity across the included studies, including variation in study design, HF subtypes, outcome definitions, and SGLT2i used, a meta-analysis was not conducted. Instead, the findings were synthesized narratively using a structured qualitative approach. Narrative synthesis was grouped thematically by HF phenotype (HFrEF or HFpEF), outcome category (mortality, hospitalization, or safety), and study design (RCT, observational, or pooled analysis) to improve interpretability. Sensitivity analysis was not performed given the qualitative design, but the implications of risk of bias were considered during narrative integration.

Publication Bias

Formal assessment of publication bias (e.g., funnel plot analysis) was not performed due to the limited number of included studies (n = 6), which limits the reliability and interpretability of such assessments.

Results

Study Selection and Data Extraction

**Figure 1:**
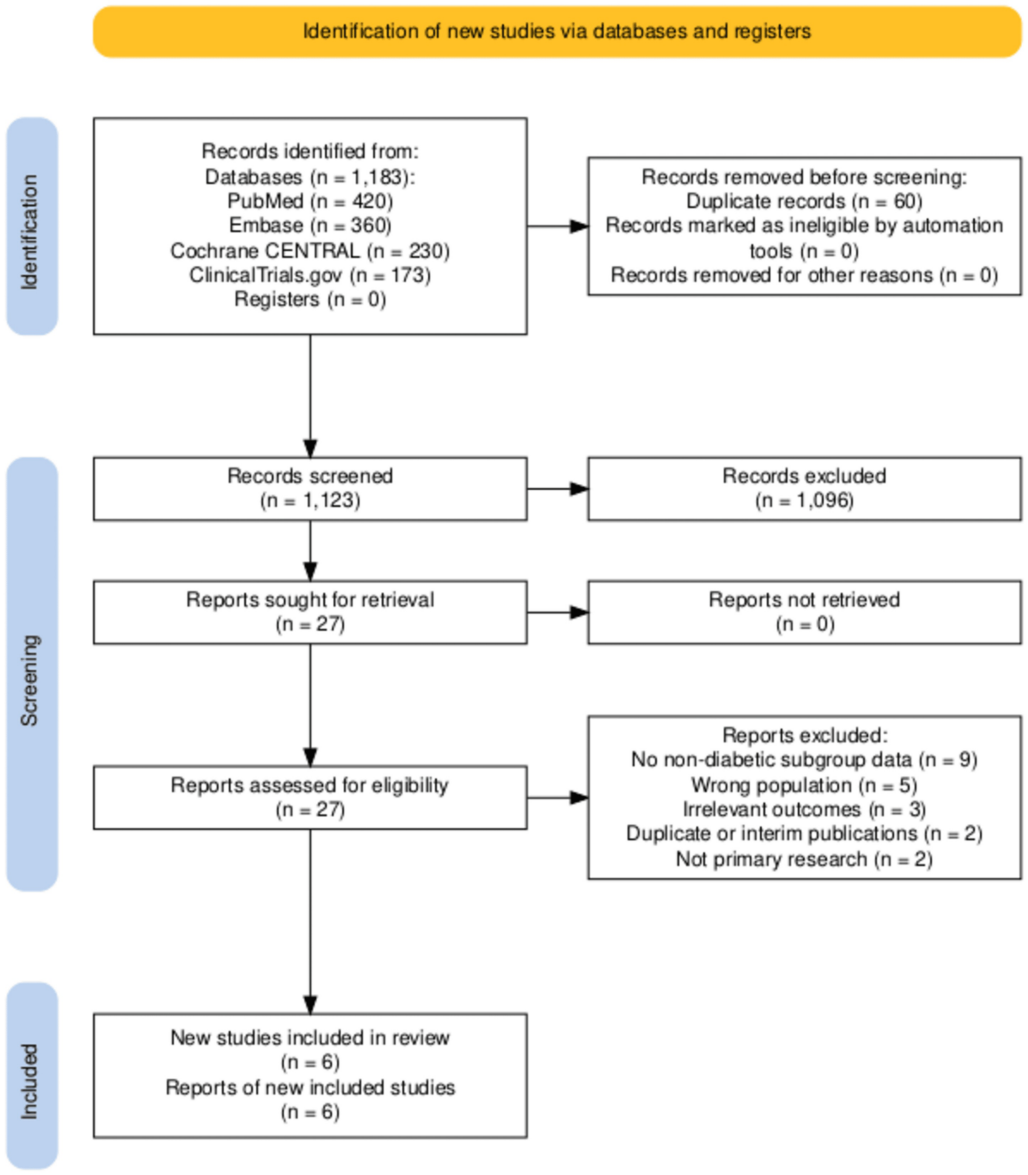
PRISMA 2020 Flow Diagram Showing the Study Selection Process for the Systematic Review PRISMA: Preferred Reporting Items for Systematic Reviews and Meta-Analyses

A total of 1,183 records were identified through database searches. Following the elimination of duplicates, a total of 1,123 titles and abstracts were evaluated. Twenty-seven full-text articles were evaluated for eligibility, with six studies fulfilling the inclusion criteria. The most common reasons for excluding the remaining full-text articles were a lack of non-diabetic subgroup data and an ineligible study design. The analysis included four RCTs, one pooled patient-level analysis of two RCTs, and one observational cohort study. The study selection process is summarized in the PRISMA 2020 flow diagram (Figure 1) [20,21]. Subgroup sizes were verified using reported baseline characteristics and supplementary material from each publication, involving 2,605 non-diabetic patients in DAPA-HF, 1,874 in EMPEROR-Reduced, 3,050 in EMPEROR-Preserved, and 84 in EMPA-TROPISM.

Study Characteristics

The characteristics of the included studies, including study design, HF subtype, sample size of non-diabetic participants, interventions, outcomes, and follow-up duration, are summarized in Table 3. Non-diabetic subgroup sizes were obtained from reported baseline characteristics or explicitly stated subgroup analyses within the original publications. Reported HRs represent adjusted values as published in the original trials (typically adjusted for age, sex, and baseline ejection fraction (EF)). Studies were conducted across North America, Europe, and multinational trial networks, supporting external validity.

**Table 3:** Summary of Included Studies Evaluating SGLT2 Inhibitors in Non-Diabetic Patients With Heart Failure RCT: randomized controlled trial; HFrEF: heart failure with reduced ejection fraction; HFpEF: heart failure with preserved ejection fraction; SGLT2i: sodium-glucose cotransporter 2 inhibitor; CV: cardiovascular; LV: left ventricular; EF: ejection fraction; HF: heart failure; NT-proBNP: N-terminal pro-B-type natriuretic peptide; QoL: quality of life; NS: not significant

Study	Design	HF Type	SGLT2i Used	Non-diabetic (n)	Key Outcomes	Follow-Up
DAPA-HF [9]	RCT	HFrEF	Dapagliflozin	2,605	↓ CV death, ↓ HF hospitalization	18.2 months
EMPEROR-Reduced [10]	RCT	HFrEF	Empagliflozin	1,874	↓ CV death, ↓ hospitalization	16 months
EMPEROR-Preserved [11]	RCT	HFpEF	Empagliflozin	3,050	↓ HF hospitalization	26.2 months
Jhund et al. (DAPA-HF + DELIVER) [25]	Patient-level pooled analysis of two RCTs	HFrEF and HFpEF	Dapagliflozin	3,656	↓ CV death, ↓ HF hospitalization	22 months
Santos-Gallego et al. [24]	RCT (EMPA-TROPISM)	HFrEF	Empagliflozin	84	↓ LV volumes, ↑ EF, ↓ NT-proBNP, improved QoL, and exercise capacity	6 months
Svanström et al. [26]	Observational	HFrEF	Dapagliflozin (79%)/empagliflozin (21%)	4,995	↓ All-cause mortality in non-diabetics; ↓ CV mortality; NS HF hospitalization	13.2 months

Risk of Bias Assessment

The risk of bias was evaluated for each included study using appropriate tools based on the study design. RCTs were assessed using the Cochrane RoB 2.0 tool, with outcomes ranging from low to moderate risk of bias (Table 1). The single observational cohort study was assessed separately using the NOS and determined to have a low risk of bias (Table 2). Risk-of-bias findings were considered during interpretation of results, but did not differentially influence study weighting.

Summary of Included Studies

This review included four RCTs and one pooled patient-level meta-analysis. The included studies were DAPA-HF, EMPEROR-Reduced, EMPEROR-Preserved, and Santos-Gallego et al. [9-11,24]. The pooled meta-analysis was conducted by Jhund et al. [25]. These trials evaluated the efficacy of SGLT2i in patients with HFrEF or HFpEF, specifically analyzing outcomes among non-diabetic subgroups. The trial by Santos-Gallego et al. focused solely on non-diabetic patients with HFrEF, whereas the patient-level meta-analysis by Jhund et al. integrated data from DAPA-HF and DELIVER, including more than 3,000 non-diabetic patients from both HFrEF and HFpEF phenotypes. To avoid duplication, DAPA-HF participants were included only once, and DELIVER data contributed exclusively through the pooled analysis. In this pooled analysis of DAPA-HF and DELIVER, dapagliflozin reduced CV death (HR 0.86; 95% confidence interval (CI) 0.76-0.97; p=0.01) and total HF hospitalizations (rate ratio 0.71; 95% CI 0.65-0.78; p<0.001), with consistent effects across both ejection-fraction phenotypes and irrespective of diabetic status. In addition, one observational cohort study was included: Svanström et al., a large Danish national database study that assessed the impact of SGLT2i on all-cause mortality in patients with HFrEF, the majority of whom (80%) were non-diabetic [26]. The study found that SGLT2i use was associated with significantly lower all-cause mortality in the non-diabetic subgroup [26]. Follow-up durations ranged from 6 to 26 months, which should be considered when comparing outcome estimates. Most participants were aged between 60 and 75 years, and baseline severity and use of concomitant HF therapies varied across studies.

Key Findings by Outcome

SGLT2i demonstrated consistent benefits across multiple clinical outcomes in non-diabetic patients with HF. Regarding CV mortality, both DAPA-HF and EMPEROR-Reduced reported significantly lower CV death among non-diabetic participants treated with SGLT2i compared to placebo, with HR of 0.82 and 0.75, respectively [9,10]. In EMPEROR-Preserved, empagliflozin also reduced HF hospitalization in the non-diabetic subgroup (HR 0.79; 95% CI 0.66-0.94; p<0.05), and the pooled analysis reported consistent effects across HFrEF and HFpEF phenotypes. Definitions of CV death and HF hospitalization were consistent with those used in each trial’s protocol. This finding was further supported by the pooled patient-level meta-analysis by Jhund et al., which combined data from DAPA-HF and DELIVER and confirmed mortality reduction across the spectrum of EF, including in non-diabetic subgroups [25]. Overall, the direction of effect was consistent across all included trials in non-diabetic subgroups.

Regarding HF hospitalizations, all included studies, including the observational cohort, consistently reported reduced hospitalization rates. The EMPEROR-Preserved trial demonstrated this effect in HFpEF patients, including those without diabetes [11]. Similarly, Jhund et al. provided robust pooled evidence of reduced hospitalizations across HF phenotypes, independent of glycemic status [25]. However, in the observational study by Svanström et al., no significant difference was observed in HF hospitalization rates among non-diabetic patients (HR 1.03, 95% CI 0.92-1.15) [26]. The different findings between EMPEROR-Preserved and the observational cohort may relate to differences in baseline disease severity and methodology.

For all-cause mortality, both DAPA-HF and the EMPEROR trials demonstrated a favorable trend, although reductions were not statistically significant in all subgroups, including non-diabetic patients [9-11]. However, the direction of effect remained consistently beneficial. Observational findings from Svanström et al. further supported this trend, showing a statistically significant reduction in all-cause mortality among non-diabetic HFrEF patients treated with SGLT2i (HR 0.73, 95% CI 0.58-0.91) [26].

In terms of safety, SGLT2i exhibited a favorable profile across all included RCTs and the observational study. In EMPA-TROPISM, changes in ventricular volumes, biomarkers, and exercise capacity represented secondary or exploratory outcomes. No increased risk of hypoglycemia, diabetic ketoacidosis (DKA), acute kidney injury (AKI), or volume depletion was observed in non-diabetic participants. No cases of ketoacidosis were reported in the non-diabetic subgroup, and although genital infections were more frequent in the SGLT2i groups compared to placebo, the available reports did not present sex-specific rates. Safety endpoints were pre-specified in all RCTs. Definitions of safety outcomes were generally consistent across RCTs, but less detailed in the observational study. Overall, the safety data support the use of SGLT2i in non-diabetic patients with HF [9-11,24,26]. Although GRADE was not applied, confidence in the evidence is supported by the low-to-moderate risk of bias across included trials.

Discussion

This systematic review provides consistent and compelling evidence that SGLT2i reduces CV events in non-diabetic patients with HF, with outcomes comparable to those seen in diabetic populations. The magnitude of benefit observed in this systematic review is broadly consistent with previous meta-analyses of SGLT2i in mixed HF populations. By integrating both observational and randomized data, this review expands the current knowledge of SGLT2i use beyond diabetic populations, providing one of the most comprehensive evaluations of their impact in non-diabetic HF across both HFrEF and HFpEF phenotypes. Based on data from four RCTs, one pooled patient-level analysis, and one large observational study, these benefits were observed across both HFrEF and HFpEF populations, including reductions in CV mortality and HF hospitalizations. Reductions in HF hospitalization and CV mortality were statistically significant in DAPA-HF, EMPEROR-Reduced, and EMPEROR-Preserved; however, not all non-diabetic subgroups reached statistical significance across every trial. Absolute event rates and number needed to treat (NNT) values were not reported consistently and, therefore, could not be included.

The findings reinforce the results of subgroup analyses from landmark trials such as DAPA-HF and EMPEROR-Reduced, both of which included a substantial proportion of non-diabetic patients [9,10]. In DAPA-HF, the HR for the primary composite outcome of worsening HF or CV death was similar in non-diabetic patients (HR 0.73; 95% CI, 0.60-0.88) compared to the overall trial population (HR 0.74; 95% CI, 0.65-0.85; p<0.001) [9]. Similarly, in EMPEROR-Reduced, empagliflozin significantly reduced the risk of the primary endpoint by 25% (HR 0.75, 95% CI 0.65-0.86; p<0.001) compared to placebo in patients with HFrEF, irrespective of glycemic status, with no evidence of interaction by glycemic status [10]. This suggests that the therapeutic mechanism of SGLT2i in HF is largely independent of their glucose-lowering effects. Heterogeneity across studies, including differences in follow-up duration, background guideline-directed medical therapy (GDMT) (e.g., ARNI and MRA use), and baseline patient characteristics, may partially explain variability in observed effects.

Mechanistically, this is supported by both clinical and preclinical data. SGLT2i induce both osmotic diuresis and natriuresis, which reduce left ventricular (LV) preload and pulmonary congestion, a key contributor to symptom burden and hospital admissions in HF [27]. They also improve myocardial energetics by shifting substrate utilization toward more energy-efficient ketone bodies, attenuate myocardial fibrosis, suppress pro-inflammatory cytokines, and promote improved vascular endothelial function [14]. Animal models have demonstrated improvements in LV remodeling and reduced oxidative stress, even in the absence of hyperglycemia [28,29]. While the observational data were adjusted for key covariates, residual confounding cannot be excluded and should be considered when interpreting real-world treatment effects.

The relevance of these mechanisms in non-diabetic HF patients is now being validated in real-world populations. The CVD-REAL Nordic registry demonstrated substantial reductions in HF hospitalization among SGLT2i users compared to other therapies, though subgroup data for non-diabetic patients were not reported separately [16]. Similarly, the EMPRISE study showed lower rates of CV death and rehospitalization with empagliflozin in HFrEF patients; however, it also lacked stratified outcome data for non-diabetic individuals [17]. In contrast, Svanström et al. provided direct evidence from a large national cohort study in Denmark, where the majority of SGLT2i users (80%) were non-diabetic [26]. The study showed that SGLT2i use was associated with a significantly lower risk of all-cause mortality among non-diabetic patients with HFrEF (HR 0.73, 95% CI 0.58-0.91), reinforcing the benefit of these agents in routine care for this population [26]. Although dapagliflozin and empagliflozin demonstrated comparable effects in the available non-diabetic subgroup analyses, direct head-to-head comparisons are lacking. Safety data in older or frail non-diabetic patients remains limited, and genital infections were more frequent in clinical trials, underscoring the need for cautious application in real-world practice.

Among RCTs, DAPA-HF and EMPEROR-Reduced reported significant reductions in CV mortality and HF hospitalization, with consistent effects among non-diabetics [9,10]. EMPEROR-Preserved extended this benefit to patients with HFpEF, including non-diabetic participants [11]. Empagliflozin reduced the risk of the composite of CV death or HF hospitalization by 21% (HR 0.79), with a consistent benefit in both non-diabetic and diabetic subgroups. Furthermore, Santos-Gallego et al. conducted an RCT that was exclusively for non-diabetic HFrEF patients, which demonstrated improvements in N-terminal pro-B-type natriuretic peptide (NT-proBNP) levels, exercise capacity, and LV remodeling [24]. This trial demonstrated significant improvements in cardiac structure and function, as well as enhanced functional capacity and quality of life, in non-diabetic patients with HFrEF treated with empagliflozin. The pooled patient-level meta-analysis by Jhund et al., which combined DAPA-HF and DELIVER, confirmed significant reductions in CV mortality and HF hospitalization across EF categories and glycemic statuses, with over 3,000 non-diabetic patients analyzed [25]. Dapagliflozin reduced CV mortality (HR 0.86), all-cause mortality (HR 0.90), and total HF hospitalizations (rate ratio 0.71), with no evidence of effect modification by EF or diabetes status [25]. Women, elderly individuals, and patients with severe renal impairment remain underrepresented in current trials, representing an important evidence gap.

The external validity of SGLT2i benefits across the HF spectrum is collectively reinforced by these data, which span diverse designs and populations. Notably, the safety profile of SGLT2i in non-diabetic HF patients was favorable in all studies [9-11,24-26]. There was no significant increase in hypoglycemia or DKA. Volume depletion and genitourinary infections were infrequent, typically mild, and manageable with standard clinical monitoring. Current guideline recommendations are generally aligned internationally, although specific guidance for non-diabetic subgroups is still limited.

Despite growing evidence, uptake of SGLT2i among non-diabetic HF patients remains variable. Surveys suggest that many clinicians still associate SGLT2i primarily with glycemic control, leading to underprescription in eligible non-diabetic individuals [30]. Furthermore, concerns about cost, lack of familiarity with class-specific side effects, and insurance restrictions may serve as additional barriers to broader use [31]. The observed renal benefits provide further rationale for early initiation of SGLT2i in non-diabetic HF, although mechanistic pathways such as enhanced ketone utilization remain largely supported by preclinical or exploratory human data.

Current guidelines from major societies are increasingly aligned with the evidence. The 2021 ESC guidelines and ACC Expert Consensus Decision Pathway now recommend SGLT2i use in all HFrEF patients, regardless of diabetic status, as part of foundational therapy alongside beta-blockers, ACEIs/ARNIs, and MRAs [19,4].

Nevertheless, it is essential to consider a number of limitations. First, most RCTs, such as DAPA-HF, EMPEROR-Reduced, and EMPEROR-Preserved, were not specifically powered for non-diabetic outcomes, despite optimistic subgroup analyses. Second, direct comparisons are confounded by the variations in baseline characteristics, endpoint definitions, and follow-up durations. Third, more dedicated research is required on this phenotype, particularly for HFpEF, which is currently underrepresented in current clinical trials. Other limitations include the absence of long-term outcome data in non-diabetic populations beyond two years. While short-to-midterm outcomes are favorable, questions remain about the durability of benefit, sustained adherence, and tolerability over the long term, especially in elderly or frail populations. Future studies should incorporate stratified analyses by HF phenotype and comorbidity profile, rather than grouping all non-diabetic patients together as a single category.

There is also growing interest in the renal-protective effects of SGLT2i, which appear to be consistent across glycemic statuses. In trials such as DAPA-CKD, reductions in progression to end-stage kidney disease (ESKD) were also observed in non-diabetic CKD patients [32]. Given the bidirectional relationship between cardiorenal dysfunction and HF, this may further justify early SGLT2i initiation in non-diabetic HF patients with reduced eGFR.

Future directions include RCTs specifically powered for non-diabetic HF populations (e.g., EMPULSE, EMPACT-MI subgroups), studies focused on HFpEF and HFmrEF phenotypes; implementation research addressing uptake, health equity, and cost-effectiveness; and mechanistic trials exploring biomarkers and structural cardiac changes in non-diabetic patients receiving SGLT2i therapy [33].

## Conclusions

In summary, the current evidence strongly supports the use of SGLT2i as a foundational treatment for HF, irrespective of diabetic status. While most of the available data are derived from subgroup analyses within randomized trials and pooled meta-analyses, rather than trials exclusively enrolling non-diabetic patients, the observed reductions in CV events and hospitalizations are consistent across phenotypes. Nevertheless, these conclusions should be interpreted in the context of heterogeneity in study populations, variations in follow-up duration, and the absence of direct head-to-head comparisons between individual agents. Importantly, some subgroups, such as older adults, women, and patients with HFpEF and advanced renal impairment, remain understudied.

From a safety standpoint, SGLT2i were generally well tolerated in non-diabetic patients, although genital infections were more frequent than placebo and required appropriate counseling. Implementation challenges such as cost, reimbursement, and limited clinician familiarity may further limit real-world uptake, despite the increasing alignment of international guidelines. Dedicated RCTs focusing specifically on non-diabetic HF populations, and incorporating stratified analyses by phenotype and comorbidity, are still needed to optimise evidence-based use of this therapeutic class.

## Appendices

Appendix A

PubMed Search Strategy

"SGLT2 inhibitors" OR dapagliflozin OR empagliflozin OR canagliflozin OR sotagliflozin AND "heart failure" OR HFrEF OR HFpEF AND non-diabetic OR "without diabetes" 

Filters applied: English language; Publication dates from January 1, 2015, to January 15, 2024.
